# A Rare Cause of Intestinal Pseudo-obstruction: Gastrointestinal Amyloid

**DOI:** 10.7759/cureus.7547

**Published:** 2020-04-05

**Authors:** Tanureet Kochar, Hamza Shah

**Affiliations:** 1 Internal Medicine, Charleston Area Medical Center / West Virginia University, Charleston, USA; 2 Gastroenterology, Charleston Area Medical Center, Charleston, USA

**Keywords:** gastrointestinal amyloidosis, intestinal obstruction, amyloid

## Abstract

Amyloidosis is characterized by extracellular deposition of the amyloid protein. It can affect multiple organ systems but it most commonly affects the heart, kidney and gastrointestinal (GI) tract. It can occur sporadically or in association with other conditions like multiple myeloma, chronic inflammatory diseases, infections etc. Its involvement of the gastrointestinal tract is rare and diffuse. It has variable manifestations in GI tract from involving the stomach to the large bowel including liver. We present a case of 55 year old Caucasian male with recent diagnosis of multiple myeloma who presented with recurrent episodes of small bowel obstruction which was later found to be caused by amyloid deposition.

## Introduction

Amyloidosis is characterized by extracellular deposition of amyloid protein. There are two most common forms of amyloidosis. The AL amyloidosis which is most commonly associated with monoclonal free light chains deposition, which was also previously called primary amyloidosis is most common. The AA amyloidosis (also called as secondary amyloidosis) is secondary to chronic inflammation in the body due to infection, rheumatoid arthritis etc. AL amyloidosis most commonly involves heart, kidney, liver and gastrointestinal tract [1]. Amyloidosis of gastrointestinal tract is rare. Small bowel involvement is the greatest when gastrointestinal tract is involved. The associated symptoms include diarrhea, hemorrhage, malabsorption, perforation and pseudo-obstruction [2,3]. 

## Case presentation

A 55 year old Caucasian male with past medical history of multiple myeloma (recently diagnosed) currently on chemotherapy with Velcade +Cytoxan and dexamethasone, chronic kidney disease, hypertension, atrial flutter s/p ablation, melanoma of the left thigh status post-surgical resection with negative lymph nodes and in remission presented to the hospital with chief complaints of multiple episodes of nausea and vomiting, diffuse abdominal pain, subjective fevers and chills from past 2 days. Last bowel movement was 2 days ago. 

**Figure 1:**
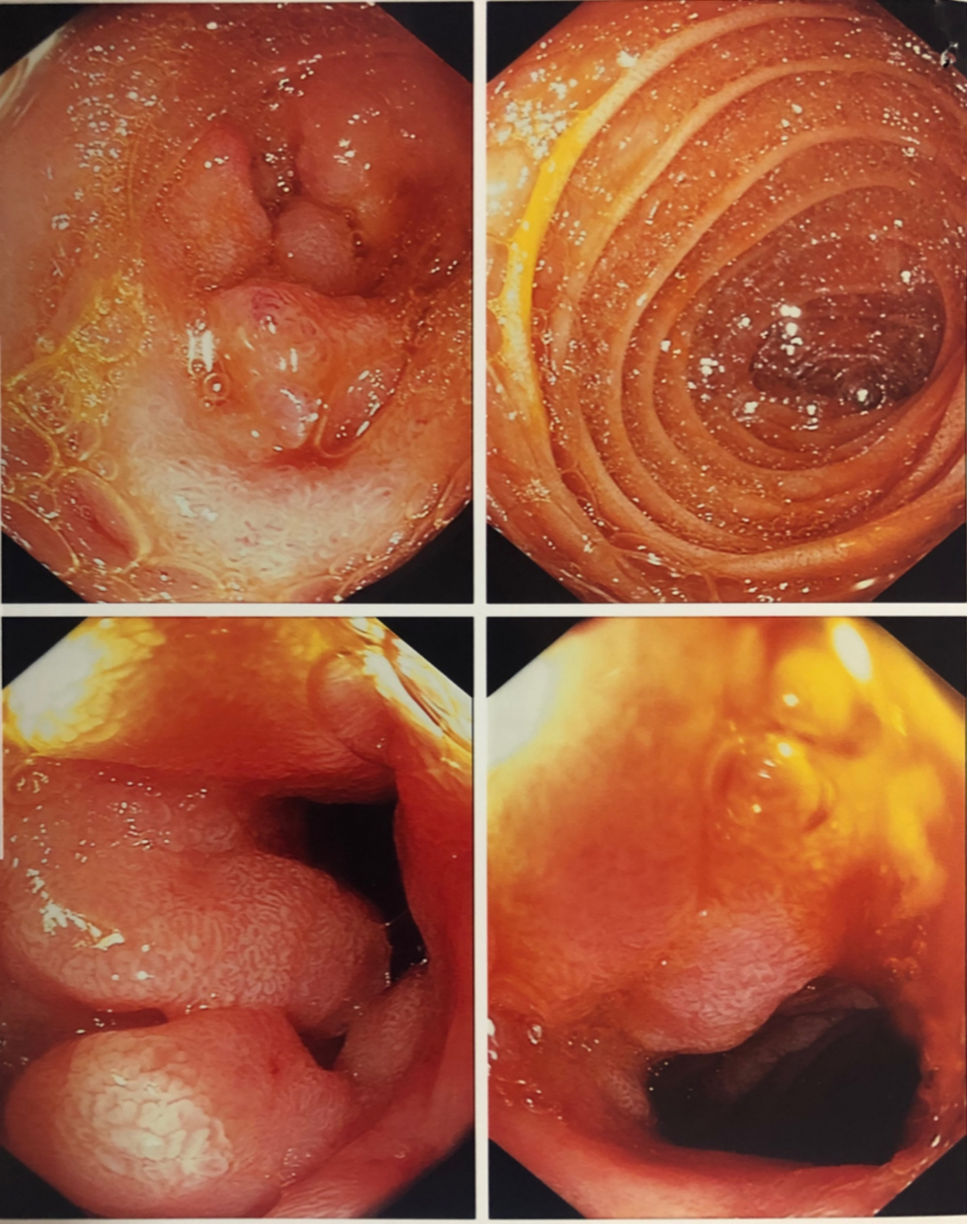
EGD revealing mild enteritis

On presentation, his vitals were stable. Labs including complete blood count, comprehensive metabolic panel was normal. CT abdomen/pelvis without contrast revealed partial small bowel obstruction. This was followed by small bowel follow through which revealed non- specific thickening of proximal small bowel loops. This was his third hospitalization with same presentation of small bowel obstruction. He was admitted one month ago and 2 weeks ago with similar presentation. During the initial hospitalization, he underwent exploratory laparotomy with no findings to suggest mechanical small bowel obstruction. Subsequently, he had an esophagogastroduodenoscopy (EGD) with push enteroscopy which identified enteritis and a Mallory-Weiss tear (Figure 1). Small bowel biopsies were performed with pathology revealing focal acute cryptitis and a background of moderate chronic nonspecific inflammation. Last colonoscopy was 9 years ago which was normal.

**Figure 2:**
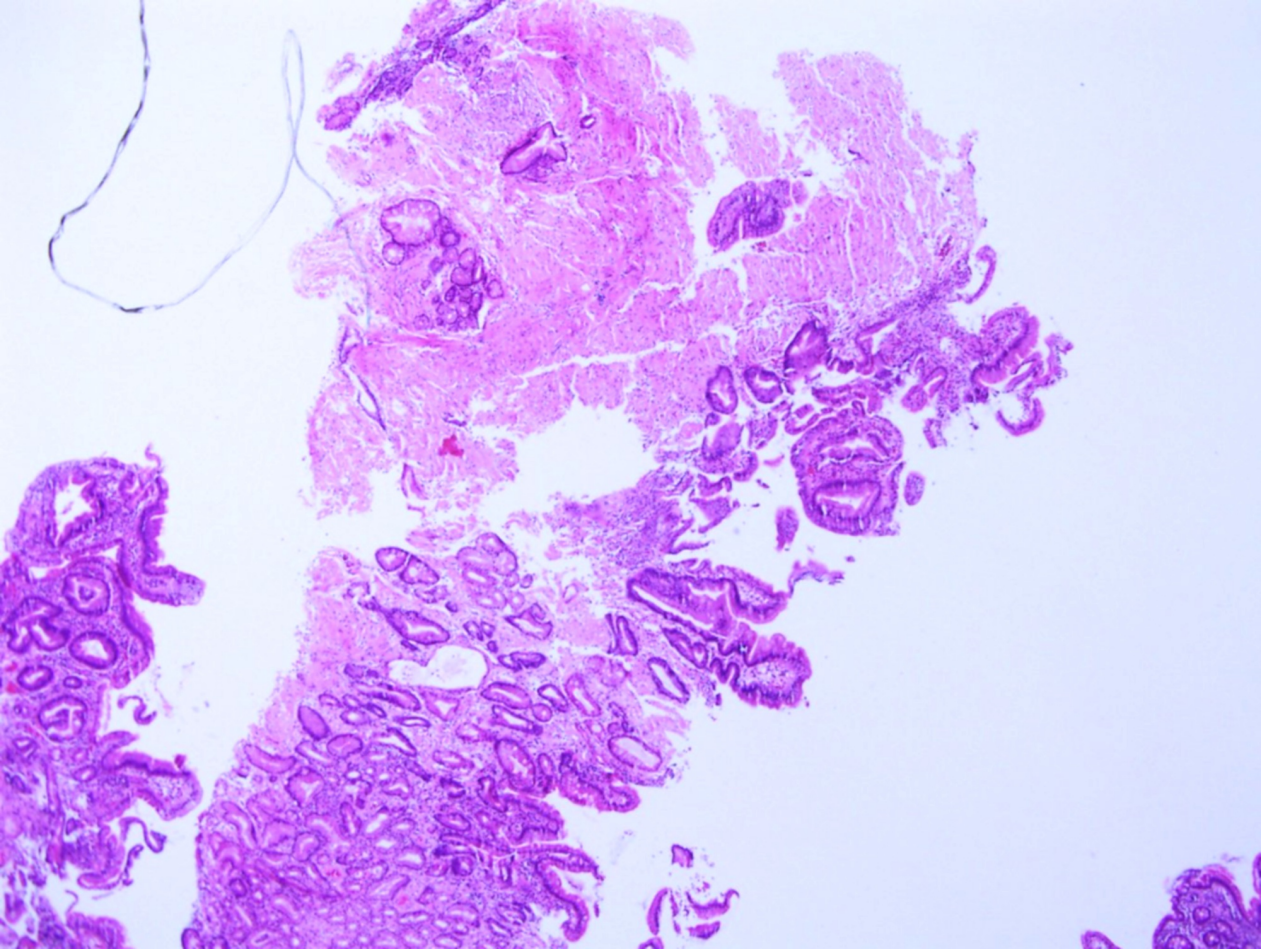
Haematoxylin and Eosin - Small Bowel Amyloid

**Figure 3:**
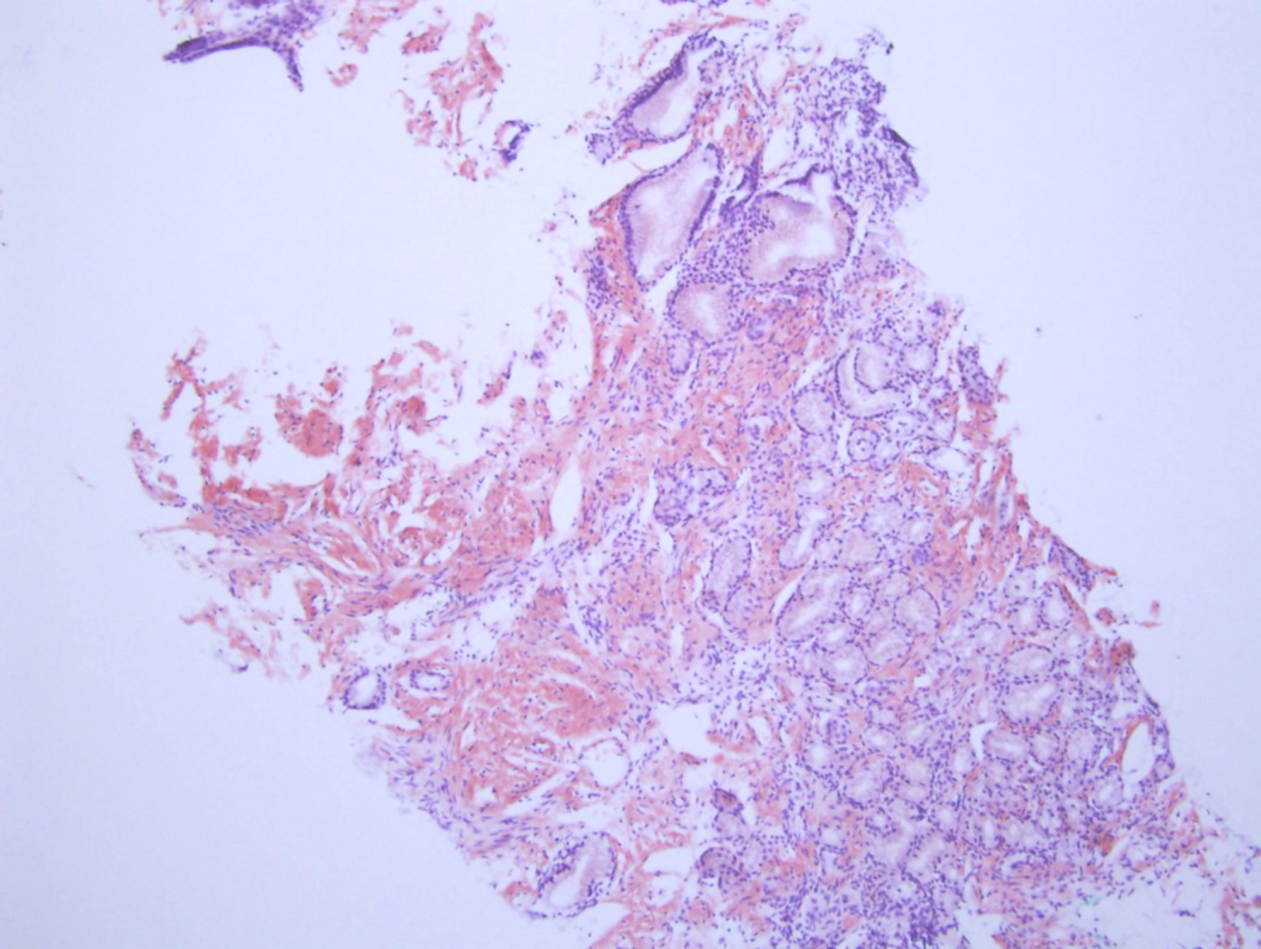
Congo Red Staining, not under polarized light revealing amyloid deposition in small bowel

**Figure 4:**
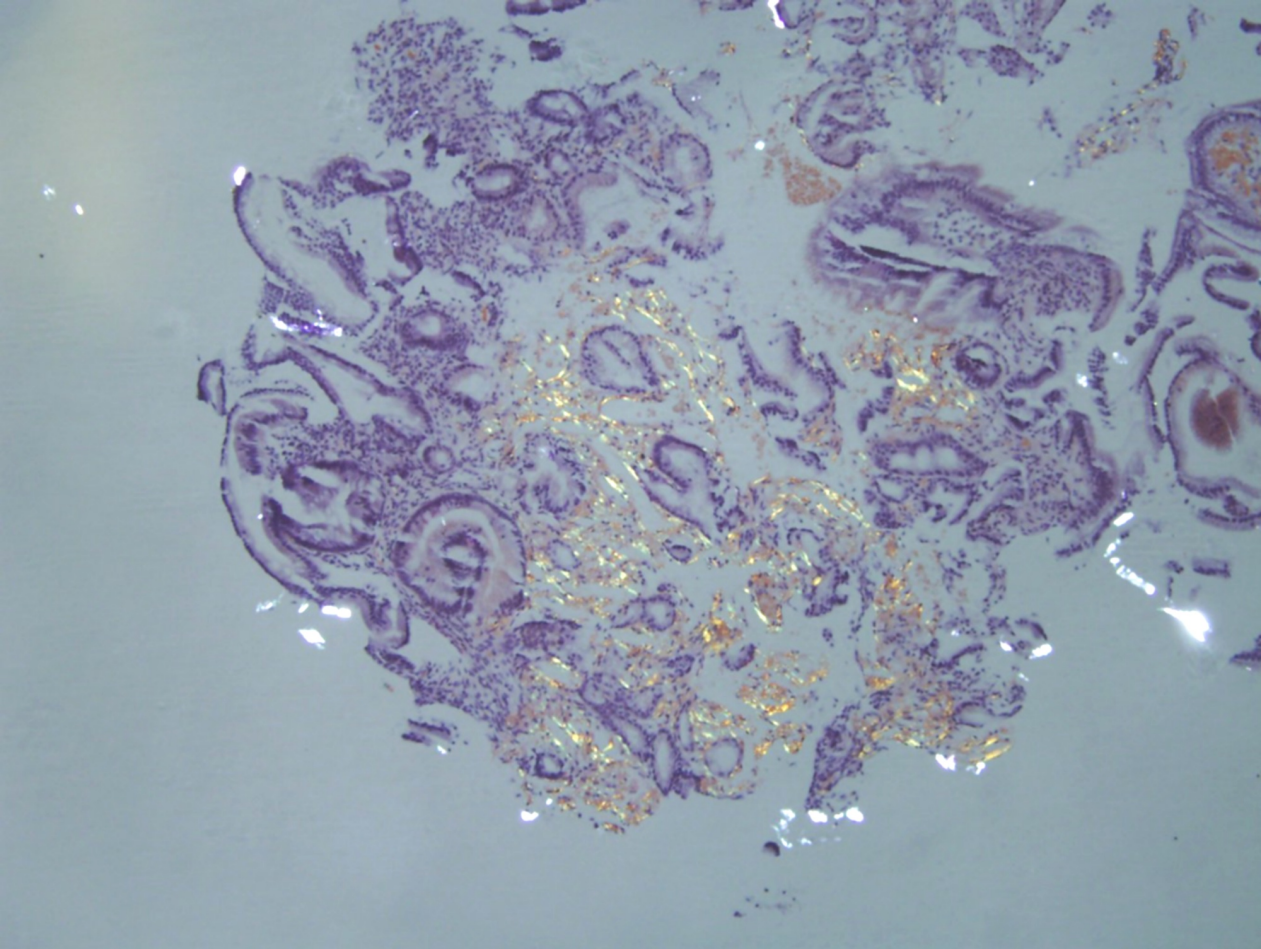
Congo red staining under polarized light revealing small bowel amyloid

On this admission, trial of nasogastric (NG) tube was given and EGD with push enteroscopy was repeated which revealed non erosive esophagitis, mild gastritis and severe duodenitis; biopsies were obtained. Congo red staining of the biopsy revealed amyloidosis of the small bowel. However, the gastric biopsy did not reveal any evidence of amyloidosis (Figure 2-4). The patient was deemed to be a good candidate for bone marrow transplant and was thus referred to a tertiary center the same. The fat pad biopsy for systemic amyloidosis was not obtained. 

## Discussion

Our case highlights the importance of the amyloidosis as a cause of intestinal pseudo -obstruction in patients with diagnosed multiple myeloma. Intestinal pseudo-obstruction is a well-known complication of amyloidosis and it should always be kept in mind in patient with multiple myeloma presenting with ileus [2].

From our review of literature, we found 33 cases of amyloidosis reported as intestinal pseudo-obstruction. Amyloidosis is characterized by extracellular deposition of abnormal protein. It involves multiple organs, most common being the heart, peripheral nerves and kidneys; however its involvement of the GI tract is mostly subclinical [3,4,5]. It has a varied presentation in gastrointestinal tract resulting from gastroparesis, nausea/vomiting, malabsorption and alteration in bowel habits among others [6]. In the GI tract, it most commonly affects the small bowel [5]. Involvement of GI tract is defined by presence of symptoms along with proof of biopsy. The above mentioned symptoms can also occur due to autonomic neuropathy due to amyloidosis which is not defined as the GI involvement of the amyloidosis [7].

Diagnosis of amyloid, irrespective of the type, depends upon clinical, laboratory and histological findings. Although endoscopic and radiological findings also assist in the diagnosis, they are very non-specific. Endoscopic findings include mucosal fragility, diffuse lesions such as ulcers, erosions, granular or plaque like mucosa of the stomach or small intestine, large duodenal folds, peripyloric ulcers and polypoid protrusions [4,8]. The radiological features of amyloidosis resemble ischemic enteritis which involves thickening of intestinal wall; however no radiological feature of amyloidosis is pathognomonic as discussed above [8]. Confirmation requires histological evaluation. The pathognomic findings are apple‐green birefringence (when viewed with crossed polarized light) and red staining (under white light microscopy) with Congo red.

No specific treatment for management of GI complications in systemic amyloidosis are available and treatment should be directed at symptomatic management. It is important to assure adequate nutrition and hydration for patients with motility disorders. Using pharmacological agents like ondansetron, granisetron, or prochlorperazine and using loperamide, lomotil for diarrhea can be helpful [9,10].

Overall, systemic amyloidosis has a very poor prognosis. The median survival in untreated patients is <2 years [1]. The most common causes of death in amyloidosis are renal failure and restrictive cardiomyopathy. Gastrointestinal complications are rare but do not generally lead to mortality, although can cause significant morbidity [11,12]. Intractable diarrhea is associated with poor survival, being 73.4% at 1 year and 38.9% at 5 years [11]. A weight loss of more than 20 pounds from the time of diagnosis is considered the important prognostic factor in terms of survival [12]. 

This work has been presented at the 2019 ACG Annual Meeting [13].

## Conclusions

The presence of amyloidosis in GI tract is rare and can have a variety of presentations. A high index of suspicion is required in patients with multiple myeloma and other chronic inflammatory conditions who present with gastrointestinal problems. In patients, without a history of systemic amyloidosis, gastrointestinal amyloidosis should be suspected in case of unexplained weight loss, autonomic dysfunction, proteinuria or malabsorption. Treatment involves treating the underlying cause. Untreated patients carry a very poor prognosis. 
